# Antibiotic Prescribing Patterns of Family Medicine Pediatric Visits: A Pharmacoepidemiological Study

**DOI:** 10.3390/healthcare13182360

**Published:** 2025-09-19

**Authors:** Reem S. AlOmar, Nouf A. AlShamlan, Ahmed M. Al-Turki, Ahmed A. Al Yateem, Abdulrahman A. Al-Abdulazeem, Najla A. Alhamed, Sameerah Motabgani, Assim M. AlAbdulkader, Abdulelah H. Almansour, Malak A. Al Shammari

**Affiliations:** 1 Department of Family and Community, College of Medicine, Imam Abdulrahman Bin Faisal University, Dammam 31441, Saudi Arabia; nashamlan@iau.edu.sa (N.A.A.); smotabgani@iau.edu.sa (S.M.); ahmansour@iau.edu.sa (A.H.A.); moalshammari@iau.edu.sa (M.A.A.S.); 2 College of Medicine, Imam Abdulrahman Bin Faisal University, Dammam 31441, Saudi Arabia; 2210000289@iau.edu.sa (A.M.A.-T.); 2210000450@iau.edu.sa (A.A.A.Y.); 2210000708@iau.edu.sa (A.A.A.-A.); 3 Pharmaceutical Affairs, King Fahad Specialist Hospital, Eastern Health Cluster, Dammam 32253, Saudi Arabia; najlaalhamed14@gmail.com

**Keywords:** epidemiology, primary care, family medicine, pharmacoepidemiology, antibiotics, prescriptions, pediatrics, public health

## Abstract

**Background/Objectives**: Understanding the medication prescribing patterns in pediatric primary care is essential for informing policy and clinical practice. In the Kingdom of Saudi Arabia (KSA), and following the 2018 antibiotic restriction policy, limited data exist on the patterns, types, and regimens of antibiotics prescribed during routine family medicine visits for children. This pharmacoepidemiological study aimed to describe the antibiotic prescribing patterns in a university-affiliated model primary healthcare center. **Methods**: A retrospective chart review was conducted for all the pediatric visits (<14 years) to general family medicine clinics between January and December 2024. Demographic characteristics, visit type, diagnosis, and antibiotic prescription details such as medication class, route, frequency, and duration were extracted from electronic medical records and analyzed descriptively. **Results:** Among the 2036 pediatric visits, 705 (34.63%) resulted in at least one prescription. Of these, 87 visits (12.34%) included an antibiotic. The most frequently prescribed antibiotic classes were nitroimidazoles (39.29%), penicillins (36.90%), and macrolides (10.71%). Penicillins were typically prescribed for 7 days twice daily as suspensions. Among the non-antibiotic prescriptions, vaccines, nutritional supplements, and analgesics were the most common. Follow-up consultations accounted for 34.09% of all the visits. **Conclusions**: A lower proportion of antibiotic prescriptions was found when compared to regional and international reports, which may reflect the impact of the antibiotic restriction policy in the country. The findings suggest a shift toward more cautious prescribing in primary care and align with the national efforts to regulate antimicrobial use. Ongoing surveillance of the prescribing trends is essential to evaluate the long-term effectiveness of these measures.

## 1. Introduction

Rational prescribing in pediatric primary care is fundamental to safe and effective health service delivery. Children are particularly vulnerable to inappropriate medication use due to age-specific pharmacokinetic differences and the increased potential for long-term consequences of early exposure to unnecessary drugs, particularly antibiotics [1]. Despite the global efforts to promote evidence-based prescribing, studies suggest that inappropriate or unnecessary prescribing remains prevalent in pediatric settings worldwide. For example, inappropriate antibiotic prescribing in children was assessed in a recent multicentric cohort study across Madagascar, Senegal, and Cambodia, which reported high rates of unnecessary prescriptions in outpatient pediatric care [2]. Similarly, in a recent Saudi study, it was reported that 30.1% of the pediatric upper respiratory tract infection cases received antibiotics, mostly without clear clinical justification, and with minimal impact from diagnostic tools such as rapid antigen detection tests and the Centor criteria [3].

In the Kingdom of Saudi Arabia (KSA), primary care serves as the initial point of contact for most pediatric patients, especially through general family medicine clinics [4]. However, data on the prescribing patterns in these settings remain limited. Of particular concern is the use of antibiotics, which account for a significant proportion of pediatric prescriptions globally and are often initiated without strong clinical indication [5]. This practice contributes to the growing threat of antimicrobial resistance, which has been recognized as a major global health concern by the World Health Organization [6].

Pharmacoepidemiological studies investigating the use of both antibiotic and non-antibiotic medications in children are crucial for understanding medication utilization trends and guiding stewardship policies. Several international studies have emphasized the importance of monitoring not only the frequency but also the type, duration, and route of administration of commonly prescribed drugs to ensure optimal pediatric care [7,8,9]. For instance, macrolides and broad-spectrum cephalosporins have frequently been implicated in inappropriate prescribing for upper respiratory infections despite established guidelines recommending conservative management in many such cases [5,10]. Moreover, topical and ophthalmic antibiotics are often used in primary care with limited microbiological confirmation, raising further concerns about unnecessary exposure [11].

In addition to antibiotics, the prescription of non-antibiotic medications such as analgesics, nutritional supplements, and asthma therapies also warrants careful evaluation. While these agents are often clinically appropriate, their widespread use, particularly without diagnostic justification, raises questions about prescribing quality and risk of polypharmacy in pediatric populations [12,13]. A recent US study analyzing pediatric outpatient prescriptions found that less than one-third met the optimal standards for both drug choice and duration [14]. Additionally, sociocultural preferences, seasonal patterns, and prescriber behavior could influence the prescribing practices in Middle Eastern settings [15,16].

The Saudi Ministry of Health implemented an antibiotic restriction policy in April 2018 [17]. Since then, antibiotics have only been prescribed with an official prescription issued by a physician, with the appropriate specialty for the diagnosis listed on the prescription. The impact of this decision must be monitored on a regular basis to improve healthcare quality. Therefore, this study aims to explore the patterns and regimens of antibiotic prescribing by family physicians, as well as the distribution of non-antibiotic medications prescribed during pediatric visits.

## 2. Materials and Methods

### 2.1. Study Design and Setting

This is a retrospective chart review study based on data from the Family and Community Medicine Centre of Imam Abdulrahman Bin Faisal University. The center is considered a model primary healthcare (PHC) center due to the availability of general family medicine clinics, as well as specialized clinics such as nutrition, diabetes, child development, well-baby clinics, and others. The center also includes its own pharmacy, laboratory, and radiology departments, and benefits from a fully digitalized medical record system.

### 2.2. Ethical Considerations

Ethical approval from the Imam Abdulrahman Bin Faisal University’s Institutional Review Board was sought (IRB-2024-01-611). The data is kept confidential and was only used for the purposes of research.

### 2.3. Study Population

The population included all visits of pediatric patients (aged < 14 years) to the family medicine clinics from the 1 January 2024 to the 31 December 2024; hence, the sample was a complete coverage of all visits during this period. Eligible visits were identified using clinic identifiers embedded within the electronic medical record system. Only visits coded as “General Family Medicine Clinics” were included. Visits linked to specialized services (e.g., urgent care, diabetes, well-baby, or nutrition clinics) were excluded at the data extraction stage.

### 2.4. Data Collection Tool

After ethical approval, the academic medical city’s IT department generated a list of all eligible pediatric visit episode numbers and exported the relevant variables from the fully digitalized medical record system into an Excel file. The variables were selected after careful review of previous studies with similar objectives while also considering the availability of those variables in the electronic medical records of patients [7,9,15]. The extracted variables included patient demographics (age, sex, and nationality), visit characteristics (visit type, month, and season), primary diagnosis categories, and medication-related data. For prescriptions, variables covered whether a medication was prescribed, antibiotic class, frequency of administration, duration, route, and dosage form. All variables extracted from the electronic health records were complete, and therefore no missing data were present in this study. Three experts (one consultant of family medicine, one clinical pharmacist, and one epidemiologist) reviewed and approved the data collection sheet.

### 2.5. Statistical Analysis

This study was descriptive in nature. All variables were categorical and described as frequencies and percentages, and no comparative or inferential statistical analyses were conducted. The variables included age categories; these included infants (<1 year old), toddlers (1 year < 3 years), preschoolers (3 years < 6 years), schoolers (6 years < 12 years), and adolescents (12 years < 14 years); sex; and nationality (Saudis vs. non-Saudis). Also, visit characteristics were included, such as the type of visit (first consultation or follow-up); month of visit; season: winter (December, January, and February), spring (March, April, and May), summer (June, July, and August), and autumn (September, October, and November); and the primary diagnosis (e.g., respiratory infections, ENT-related conditions, and allergic and skin conditions). Medication-related variables included the classes of antibiotics, as well as the frequency, duration in days, route, and dosage form. The STATA statistical software version 15.0 (Stata Corporation) was used for the analysis [18].

## 3. Results

### 3.1. Sociodemographic and Visit Characteristics of Pediatric General Family Medicine Clinics

The total number of pediatric visits during the study period was 2036, 52.9% of which were for female patients. Visits of school-aged children were the most common (43.5%), and the least common were for infants (5.1%). Saudi nationals comprised most visits (70.6%) (Table 1).

Table 2 presents the visit characteristics, where first consultation visits were higher than follow-ups (65.9% and 34.1%, respectively). Most visits occurred during spring, followed by autumn. With regard to the primary diagnoses, over half the visits were for lab-result readings or routine check-ups (58.6%), followed by nutritional deficiencies (9.1%), allergic and skin conditions (8.3%), and respiratory infections (6.0%).

### 3.2. Prescribing Patterns of Pediatric Visitors Regarding General Family Medicine

Table 3 describes the number of visits with a valid prescription. The results show that, of the total number of visits within the study period, seven-hundred-five (34.6%) included a prescription regardless of the type of medication. Of those seven-hundred-five visits with a prescription, 12.3% were prescribed an antibiotic. Only three visitors were prescribed a combination of two antibiotics.

Table 4 presents the classes of antibiotics according to the age and sex of pediatric patients. A total of eighty-seven antibiotic prescriptions were issued to eighty-four pediatric patients, with three patients receiving two different antibiotic classes. This accounts for the cumulative percentage exceeding 100% across classes. Among those pediatric patients who received antibiotic prescriptions, nitroimidazoles were the most frequently prescribed class, constituting 39.3% of all the prescriptions. This was followed by penicillins at 36.9% and macrolides at 10.7%. Other less commonly prescribed classes included fluoroquinolones, lincosamides, folate pathway inhibitors, and aminoglycosides. None of the patients were prescribed antibiotics in the cephalosporin, tetracycline, or sulfonamide classes. Penicillins were more commonly prescribed to male patients (39.3%) compared to females (35.7%). Males also had a higher proportion of macrolides (14.3% vs. 8.9%) and nitroimidazoles (46.4% vs. 35.7%). On the other hand, fluoroquinolones, lincosamides, aminoglycosides, and folate pathway inhibitors were prescribed exclusively or more frequently to females. The age-specific prescribing patterns showed that penicillins were the most commonly prescribed antibiotic class among preschoolers (61.1%), whereas nitroimidazoles were more commonly prescribed for toddlers (58.3%). When stratified by sex, male preschoolers most received penicillins (66.7%), while male toddlers and infants exclusively received nitroimidazoles (100%). In females, penicillins were most frequently prescribed for preschoolers (58.3%) and school-aged children (47.8%), whereas nitroimidazoles were most frequent among adolescents (46.7%).

Table 5 shows the classes of antibiotics according to the prescribing regimen. In terms of frequency of administration, nearly all the penicillins (96.8%) were prescribed twice daily, with only one prescription specified once daily. All the macrolides (100%) were prescribed once daily, whereas nitroimidazoles were mostly once daily (48.5%). Regarding the duration of therapy, penicillins were mostly prescribed for 7 days (74.2%), with smaller proportions for 10 days, 14 days, and 5 days. Macrolides were typically prescribed for 5 days (66.7%), while fluoroquinolones were split between 5 and 7 days. Nitroimidazoles displayed the widest variation in duration, with regimens ranging from 2 to 60 days. In terms of route of administration, oral antibiotics dominated prescriptions for penicillins, macrolides, and folate pathway inhibitors. In contrast, fluoroquinolones were exclusively administered via the ophthalmic route, and lincosamides were primarily topical. The dosage form patterns reflected these routes. Eye drops were used exclusively for fluoroquinolones (100%), and lincosamides were entirely prescribed as ointments or gels (100%). Nitroimidazoles displayed a broad distribution, where 9.1% were suspensions, 18.2% drops, and 54.6% ointments/gels.

### 3.3. Distribution of Diagnoses Requiring a Prescription and the Medications Prescribed

**Figure 1:**
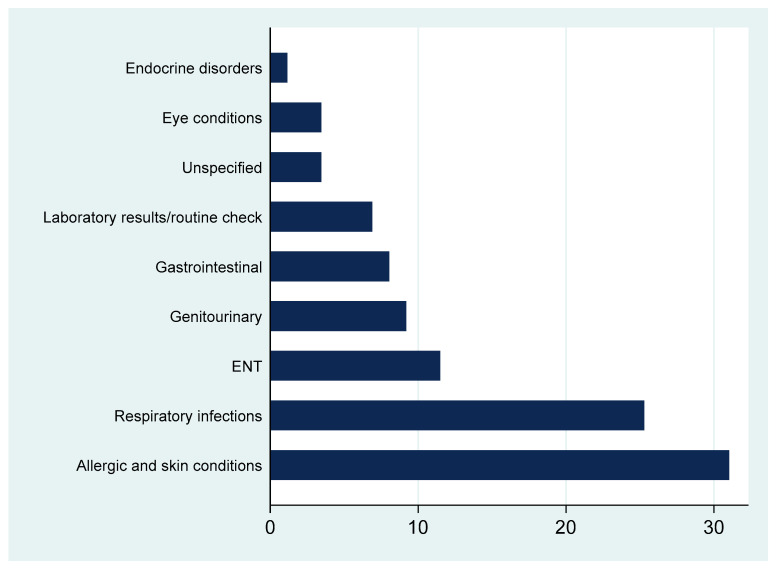
Percentages of recorded diagnoses requiring an antibiotic.

Figure 1 shows that dermatological and respiratory conditions accounted for the largest share of antibiotic prescriptions, followed by ENT, genitourinary, and gastrointestinal diagnoses. Fewer prescriptions were linked to routine check-ups or unspecified complaints.

**Figure 2:**
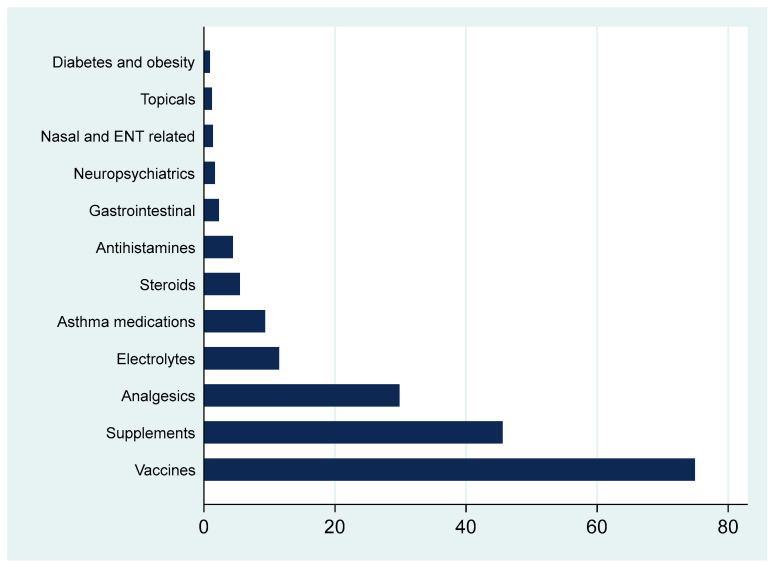
Percentages of all other non-antibiotic medications prescribed by a pediatric family medicine clinic. Note that percents exceed 100% as one patient may have multiple medications.

As shown in Figure 2, the most commonly prescribed non-antibiotic medications were vaccines, nutritional supplements, and analgesics. Other categories such as electrolyte replacements, asthma therapies, and gastrointestinal agents were less frequent.

## 4. Discussion

This pharmacoepidemiological study offers critical insights into antibiotic and non-antibiotic prescribing patterns among pediatric patients attending general family medicine clinics in the KSA. By leveraging a complete year of digitalized prescribing data, the study provides real-world evidence on the current practices within the post-antibiotic-policy era in the country. The inclusion of detailed regimen characteristics (e.g., duration, route, and dosage form) adds depth to conventional surveillance approaches. Moreover, the stratification by age and sex allows for nuanced interpretation of potential prescribing biases or trends, which are seldom reported in the regional literature.

The study observed a higher frequency of school-aged children presenting to family medicine clinics compared to younger children. This partially aligns with the existing literature, which reports varied age distributions in pediatric primary care [4,19]. Additionally, mandatory school health screenings may contribute to increased referrals to family medicine clinics as these screenings often identify health concerns requiring further evaluation and management in a primary care setting.

Over one-third of the visits were found to be follow-ups. This proportion aligns with international evidence suggesting that follow-up care constitutes a significant component of pediatric primary care utilization, often driven by chronic conditions, reassessment of symptoms, or review of laboratory findings [20]. In the Saudi context, such follow-up visits may reflect an increasing emphasis on continuity of care and parental preference for ongoing monitoring, even in cases that may not strictly require reassessment. While this may indicate improved care engagement and provider accessibility, it also highlights the need to evaluate the clinical appropriateness of follow-up scheduling [21]. This is also indicated within the current results where over half the visits were due to routine check-ups. The mere fact that this was documented in this way as a diagnosis is problematic and suggests a need for quality improvement in documentation as vague labels may obscure morbidity, impede epidemiological tracking, and reduce data comparability.

With regard to prescription patterns, approximately one-third of the visits included at least one prescription, reflecting a high reliance on pharmacologic management in primary care. While this is lower than the rates reported in other primary care settings internationally, it still reflects a notable reliance on pharmacologic management within family medicine clinics [1,14]. While many prescriptions may be clinically appropriate, such a pattern also raises questions about potential overmedicalization, particularly the frequency of prescriptions issued during routine check-ups and for conditions that may often be self-limiting. In contexts where diagnostic uncertainty or parental expectations influence prescribing behavior, there is a risk that pharmacologic solutions may be prioritized over non-pharmacologic strategies such as reassurance, monitoring, or behavioral advice [8]. These observations emphasize the importance of promoting evidence-based prescribing, enhancing shared decision-making, and reinforcing the role of non-drug interventions in the management of common childhood conditions in family medicine.

Interestingly, only 12.34% of the visits with a prescription involved antibiotics, reflecting a cautious approach to antibiotic use. This contrasts with findings from other primary healthcare settings, where antibiotic prescribing exceeded “rational use standards” and antibiotic use along with complementary testing were significantly associated with medical overuse [7,22]. Furthermore, only three visits involved the prescription of a combination of two antibiotics, underscoring a conservative approach to antibiotic therapy in this population. This contrasts sharply with the findings from other studies, where the proportions of antibiotics prescribed were high, in some places reaching 43% [7,9,23]. The overwhelmingly low prescribing patterns in our setting may be due to the adherence of family physicians to the antibiotic restriction policy that was introduced in April of 2018 [17]. Overall, these findings highlight a balanced approach between the need for pharmacological treatment and the cautious use of antibiotics in clinical decision-making. This is particularly important given that inappropriate prescribing, such as the use of antibiotics for viral illnesses or for incorrect durations, remains a significant concern in many healthcare settings (6).

Exploration of the classes of antibiotics has shown that, in this cohort, a narrow prescribing spectrum is dominant, with penicillins and nitroimidazoles accounting for the majority of the prescriptions. A key finding was the complete absence of cephalosporins, sulfonamides, and tetracyclines, which are frequently used in pediatric populations elsewhere. For instance, cephalosporins ranked amongst the most commonly prescribed antibiotics in Hungary, Ethiopia, and Australia, often for respiratory and urinary tract infections [9,24,25]. The absence of these classes in our setting likely reflects local formulary limitations, adherence to the national antibiotic restrictions introduced in 2018, and possibly a greater emphasis on narrow-spectrum prescribing in Saudi primary care [17].

The use of penicillins was consistent with the clinical expectations, mostly prescribed to preschool-aged children, formulated primarily as suspensions, and prescribed twice daily over 7 days. This pattern suggests alignment with standard treatment of common pediatric infections, such as otitis media, streptococcal pharyngitis, and non-complicated lower respiratory tract infections [26,27]. By contrast, nitroimidazole prescribing presented a more complex picture. Although their high frequency may initially appear concerning, particularly since these agents are primarily indicated for anaerobic or protozoal infections, a deeper review revealed that many were topical or ophthalmic formulations, appropriate for localized skin or mucosal infections. However, the wide variation in prescribed duration (2 to 60 days) and the absence of recorded indications emphasize the need for improved documentation and diagnostic linkage. While prolonged courses may have been clinically justified in a minority of cases, such prescribing patterns highlight the need for closer monitoring, clearer diagnostic documentation, and further research on the safety and appropriateness of nitroimidazole use in pediatric primary care. Similar gaps in prescription justification have been highlighted in international studies, including Dutch and Australian primary care datasets, which reported inconsistencies between guideline recommendations and class-level prescribing [25,28].

Another notable finding was the sex-based difference in antibiotic class distribution. Male patients were prescribed a narrower range, largely penicillins and nitroimidazoles, while female patients, particularly school-aged and adolescent females, were prescribed a broader array of classes, including fluoroquinolones, aminoglycosides, lincosamides, and folate pathway inhibitors. These trends may reflect differences in clinical presentations (e.g., ophthalmic infections or UTIs), but they also raise questions about prescriber patterns and consistency. A study in Jordan found similar age and sex influences on prescribing behavior, whereas data from Abu Dhabi suggested more uniform prescribing across genders [8,29].

Upon closer assessment of the indications of antibiotic prescribing, dermatological and respiratory complaints were the predominant drivers of antibiotic use. This pattern aligns with the global trends indicating that skin and respiratory infections are frequently cited indications for antibiotics in children despite the high likelihood of non-bacterial etiology in many cases [30,31]. Furthermore, the relatively frequent use of antibiotics for dermatological complaints may be attributed to diagnostic uncertainty or caregiver expectations, both of which are known to influence physician decision-making in pediatric settings [32]. Although some skin conditions, such as impetigo or infected eczema, may warrant antibiotic treatment, others, especially those of allergic origin, do not. Similarly, most pediatric respiratory infections are viral and self-limiting, making antibiotics unnecessary in many cases [33]. In such contexts, prescribing behavior may be influenced by diagnostic uncertainty as distinguishing bacterial from viral illness is often difficult, as well as by parental expectations, which previous studies have reported as important drivers of medication use in pediatric primary care [34,35]. The prescribing patterns observed highlight the need for decision support tools and educational initiatives that encourage evidence-based differentiation between bacterial and viral infections to strengthen the antimicrobial stewardship (AMS) practices within primary care.

Our findings highlight a strong focus on preventive care, with non-antibiotic prescriptions most often administered for vaccines and nutritional supplements. This is in line with the national and international guidelines encouraging routine immunizations and addressing micronutrient deficiencies among children [36,37]. Indeed, the high proportion of preventive care prescriptions, along with the relatively low antibiotic prescribing rate, may indicate a favorable shift toward proactive guideline-based pediatric care. These patterns support the value of family medicine models that integrate preventive services with acute care, providing a foundation for reducing antimicrobial resistance and enhancing population health outcomes in the pediatric population [38].

### 4.1. Implications for Primary Care Practice and Policy

Although the overall rate of antibiotic prescribing in this study was low compared to regional and international reports, it cannot be assumed that all the prescriptions were clinically appropriate. Some prescriptions may still have been unnecessary or of prolonged duration despite the national antibiotic restriction policy. This policy is implemented through several components, most notably the enforcement of a prescription-only requirement, formulary restrictions that limit the availability of broad-spectrum agents in primary care, and pharmacist oversight prior to dispensing [39]. The lack of consistent clinical detail in the electronic records precluded a formal appropriateness assessment; however, these findings underscore the need for future studies to evaluate antibiotic use against guideline-based criteria and to strengthen the audit and feedback mechanisms in family medicine clinics.

From a pharmacoepidemiological standpoint, these findings emphasize the importance of monitoring not only the frequency but also the class, route, dosage form, and duration of antibiotics prescribed. The frequent use of non-systemic formulations, such as topical lincosamides and ophthalmic fluoroquinolones, reflects a dimension of antibiotic exposure that is often underreported yet relevant to resistance development. Meanwhile, from a public health perspective, our data supports the expansion of AMS programs into primary care, a sector that is frequently overlooked in favor of hospital-based interventions. The findings support targeted AMS strategies tailored to family medicine clinics, such as prescriber education, point-of-care diagnostic tools, clearer indication documentation, and routine audit–feedback cycles. Locally adapted guidelines that reflect common prescribing scenarios in general practice could reduce class-level variation and promote rational use of antimicrobials in pediatric patients.

### 4.2. Strengths and Limitations

This study provides a comprehensive analysis of the pediatric prescribing patterns in a model PHC using a full year of digital records, stratified by age, sex, diagnosis, and regimen details. It is among the few Saudi-based studies to explore both antibiotic and non-antibiotic use in general family medicine settings. However, the study was conducted in a single university-affiliated model PHC center, which benefits from integrated electronic records, a multidisciplinary team, and structured prescribing policies. While this setting provides valuable insights into the prescribing patterns within an academic and resource-rich environment, the findings may not be generalizable to other primary care contexts in Saudi Arabia, particularly rural facilities or private sector clinics where resources, patient populations, and prescribing behaviors may differ substantially. A further limitation relates to diagnostic accuracy. More than half of the visits were coded as “routine check-up” and a smaller proportion as “unspecified”. Such vague labels may reduce the validity of interpreting prescribing appropriateness as the true clinical context of some prescriptions could not be fully ascertained. Another important consideration is that the study was not designed to evaluate the clinical appropriateness of antibiotic prescribing against guidelines since detailed clinical indicators, such as microbiological confirmation and weight, were not consistently available in the electronic records and therefore could not be analyzed. Although the descriptive nature of the study enabled complete data capture and detailed descriptions of the prescribing patterns, it precluded comparative statistical analyses across categories. Expanding future studies to include additional years of data, as well as incorporating prescriber rationale and/or appropriateness of therapy, would provide opportunities for temporal comparisons and thereby increase clinical relevance.

## 5. Conclusions

This study offers a detailed account of the pediatric prescribing patterns in a model family medicine setting, revealing both the scope and structure of medication use. The most commonly prescribed antibiotic classes, nitroimidazoles, penicillins, and macrolides, were consistently issued as oral suspensions, with the durations typically ranging from 5 to 7 days and presenting standardized frequency patterns. Such uniformity suggests the presence of established prescribing routines. Importantly, the overall rate of antibiotic prescribing was lower than what has been reported in similar regional and international contexts; however, these findings should be interpreted strictly within the scope of prescribing patterns as assessment of appropriateness was beyond the study’s objectives. This trend may reflect the influence of national policies restricting antibiotic dispensing without medical supervision and may indicate a shift toward more conservative use within primary care. As the Ministry of Health continues to promote rational prescribing, these findings underscore the value of monitoring prescription behavior in real-world settings to assess the long-term impact of policy measures and evolving clinical norms.

## Figures and Tables

**Table 1:** Sociodemographic characteristics of pediatric visitors to general family medicine clinics in 2024.

Characteristics	N (%) 2036 (100.00)
**Sex**	
Males	959 (47.10)
Females	1077 (52.90)
**Age group**	
Infants	103 (05.05)
Toddlers	274 (13.46)
Preschoolers	395 (19.40)
Schoolers	886 (43.52)
Adolescents	378 (18.57)
**Nationality**	
Saudi	1438 (70.63)
Non-Saudi	598 (29.37)

**Table 2:** Characteristics of pediatric visits to general family medicine clinic in 2024.

Characteristics	N (%) 2036 (100.00)
**Type of visit**	
First consultation	1342 (65.91)
Follow-up	694 (34.09)
**Season**	
Spring	551 (27.06)
Autumn	543 (26.67)
Summer	510 (25.05)
Winter	432 (21.22)
**Primary diagnosis**	
Routine check-up	1193 (58.60)
Nutritional deficiencies	186 (09.14)
Allergic and skin conditions	168 (08.25)
Respiratory infections	123 (06.04)
Genitourinary and puberty-related	66 (03.24)
Gastrointestinal	57 (02.80)
Musculoskeletal and orthopedics	53 (02.60)
Developmental, psychiatric and behavioral	48 (02.36)
Endocrine disorders and diabetes	41 (02.01)
Unspecified (e.g., general fatigue)	36 (01.77)
ENT	27 (01.33)
Eye conditions	21 (01.03)
Neurological disorders	17 (00.83)

**Table 3:** Prescription patterns of pediatric visits to general family medicine clinic in 2024.

Visits with Prescriptions	N (%) 2036 (100.00)
**Visit resulted in prescribed medications**	
No	1331 (65.37)
Yes	705 (34.63)
**If yes, antibiotics prescribed? (n = 705)**	
No	618 (87.66)
Yes	87 (12.34)
**Combined antibiotics (n = 705)**	
Single	81 (96.43)
Two antibiotics prescribed	3 (03.57)

**Table 4:** Antibiotic classes according to age and sex of pediatric patients.

Age and Sex Characteristics ^¥^	Penicillins N (%) 31 (36.90)	Macrolides N (%) 9 (10.71)	Fluoroquinolones N (%) 5 (05.95)	Aminoglycosides N (%) 2 (02.38)	Lincosamides N (%) 4 (04.76)	Nitroimidazoles N (%) 33 (39.29)	Folate Pathway Inhibitors N (%) 3 (03.57)
**Males (n = 28)**	11 (39.29)	4 (14.29)	1 (03.57)	0	0	13 (46.43)	0
**Females (n = 56)**	20 (35.71)	5 (08.93)	4 (07.14)	2 (03.57)	4 (07.14)	20 (35.71)	3 (05.36)
**All age groups**							
Infants (n = 1)	0	0	0	0	0	1 (100.00)	0
Toddlers (n = 12)	2 (16.67)	2 (16.67)	1 (08.33)	0	0	7 (58.33)	0
Preschoolers (n = 18)	11 (61.11)	3 (16.67)	1 (05.56)	1 (05.56)	0	2 (11.11)	0
Schoolers (n = 33)	15 (45.45)	2 (06.06)	2 (06.06)	0	1 (03.03)	14 (42.42)	1 (03.03)
Adolescents (n = 20)	3 (15.00)	2 (10.00)	1 (05.00)	1 (05.00)	3 (15.00)	9 (45.00)	2 (10.00)
**Males**							
Infants (n = 1)	0	0	0	0	0	1 (100.00)	0
Toddlers (n = 6)	0	0	0	0	0	6 (100.00)	0
Preschoolers (n = 6)	4 (66.67)	1 (16.67)	0	0	0	1 (16.67)	0
Schoolers (n = 10)	4 (40.00)	2 (20.00)	1 (10.00)	0	0	3 (30.00)	0
Adolescents (n = 5)	3 (60.00)	1 (20.00)	0	0	0	2 (40.00)	0
**Females**							
Toddlers (n = 6)	2 (33.33)	2 (33.33)	1 (16.67)	0	0	1 (16.67)	0
Preschoolers (n = 12)	7 (58.33)	2 (16.67)	1 (08.33)	1 (08.33)	0	1 (08.33)	0
Schoolers (n = 23)	11 (47.83)	0	1 (04.35)	0	1 (04.35)	11 (47.83)	1 (04.35)
Adolescents (n = 15)	0	1 (06.67)	1 (06.67)	1 (06.67)	3 (20.00)	7 (46.67)	2 (13.33)

^¥^ Three patients received two antibiotic classes; therefore, percentages exceed 100%.

**Table 5:** Antibiotic classes according to prescribing regimens for pediatric patients.

Age and Sex Characteristics ^¥^	Penicillins N (%) 31 (36.90)	Macrolides N (%) 9 (10.71)	Fluoroquinolones N (%) 5 (05.95)	Aminoglycosides N (%) 2 (02.38)	Lincosamides N (%) 4 (04.76)	Nitroimidazoles N (%) 33 (39.29)	Folate Pathway Inhibitors N (%) 3 (03.57)
**Daily frequency**							
One time	1 (03.23)	9 (100.00)	1 (20.00)	1 (50.00)	1 (25.00)	16 (48.48)	0
Two times	30 (96.77)	0	2 (40.00)	1 (50.00)	3 (75.00)	11 (33.33)	3 (100.00)
Three times	0	0	0	0	0	6 (18.18)	0
Four times	0	0	2 (40.00)	0	0	0	0
**Duration in days**							
2	0	0	0	0	0	2 (6.06)	0
3	0	2 (22.22)	0	0	0	1 (03.03)	0
5	1 (03.23)	6 (66.67)	3 (60.00)	1 (50.00)	0	0	2 (66.67)
6	0	0	0	0		1 (03.03)	0
7	23 (74.19)	0	2 (40.00)	1 (50.00)	0	0	0
10	4 (12.90)	0	0	0	0	3 (09.09)	0
14	3 (09.68)	1 (11.11)	0	0	0	4 (12.12)	0
30	0	0	0	0	2 (50.00)	30 (30.30)	1 (33.33)
60	0	0	0	0	2 (50.00)	2 (06.06)	0
**Route**							
Oral	31 (100.00)	9 (100.00)	0	0	0	9 (27.27)	3 (100.00)
Ophthalmic/otic	0	0	5 (100.00)	1 (50.00)	0	6 (18.18)	0
Topical	0	0	0	1 (50.00)	4 (100.00)	18 (54.55)	0
**Dosage form**							
Suspension	29 (93.55)	7 (77.78)	0	0	0	3 (09.09)	1 (33.33)
Tablets	2 (06.45)	2 (22.22)	0	0	0	6 (18.18)	2 (66.67)
Eye/ear drops	0	0	5 (100.00)	1 (50.00)	0	6 (18.18)	0
Ointment/Gel	0	0	0	1 (50.00)	4 (100.00)	18 (54.55)	0

^¥^ Three patients received two antibiotic classes; therefore, percentages exceed 100%.

## Data Availability

The data supporting the findings of this study are available from the corresponding author upon reasonable request.

## Abbreviations

The following abbreviations are used in this manuscript:

KSA	Kingdom of Saudi Arabia
PHC	Primary Healthcare Center

## References

[1] Clavenna A., Bonati M. (2009). Drug prescriptions to outpatient children: A review of the literature. Eur. J. Clin. Pharmacol..

[2] Ardillon A., Ramblière L., Kermorvant-Duchemin E., Sok T., Zo A.Z., Diouf J.B., Long P., Lach S., Sarr F.D., Borand L. (2023). Inappropriate antibiotic prescribing and its determinants among outpatient children in 3 low- and middle-income countries: A multicentric community-based cohort study. PLoS Med..

[3] Al-Baghli N.A., Al Saif A.Z., A Al Dorazi S., Zainaldeen M.H., Alameer A.H., Albaghli S., Al-Dawood A.M., Buhelaiga S.M., Alsalim B.S., A Rabaan A. (2023). Antibiotic-Prescribing Patterns Among Patients With Respiratory Symptoms in the Eastern Province, Kingdom of Saudi Arabia. Cureus.

[4] Yousef H.A., Wahab M.M.A., Alsheikh S., Alghamdi R., Alghamdi R., Alkanaan N., Al-Qahtani M., Albuali W.H., Almakhaita H., Aldossari M. (2022). Characteristics of Pediatric Primary Healthcare Visits in a University-Based Primary Healthcare Center in Saudi Arabia. Children.

[5] Hersh A.L., Jackson M.A., Hicks L.A. (2013). Principles of judicious antibiotic prescribing for upper respiratory tract infections in pediatrics. Pediatrics.

[6] World Health Organisation (2016). Global Action Plan on Antimicrobial Resistance.

[7] Jahan S., Al-Saigul A.M., Hamdelsseed S.A. (2019). Primary health care physicians’ prescribing patterns for children under five in Qassim, Saudi Arabia. Prim. Health Care Res. Dev..

[8] Al-Shatnawi S.F., Al-Hosban S.Y., Altawalbeh S.M., Khasawneh R.A. (2021). Antibiotic prescribing patterns for childhood infections in ambulatory settings in Jordan. Int. J. Clin. Pract..

[9] Galistiani G.F., Benkő R., Babarczy B., Papp R., Hajdu Á., Szabó É.H., Viola R., Papfalvi E., Visnyovszki Á., Matuz M. (2022). Prescribing Patterns and Variations of Antibiotic Use for Children in Ambulatory Care: A Nationwide Study. Antibiotics.

[10] Vaz L.E., Kleinman K.P., Raebel M.A., Nordin J.D., Lakoma M.D., Dutta-Linn M.M., Finkelstein J.A. (2014). Recent trends in outpatient antibiotic use in children. Pediatrics.

[11] Cherry M.D., Tapley A., Quain D., Holliday E.G., Ball J., Davey A., van Driel M.L., Fielding A., Spike N., FitzGerald K. (2021). Antibiotic prescribing patterns of general practice registrars for infective conjunctivitis: A cross-sectional analysis. J. Prim. Health Care.

[12] Park B., Lee H., Choi H., Lee J. (2024). Age-related off-label drug prescribing in pediatric patients in South Korea and consistency of labeling compared to the United States, Europe, and Japan. Clin. Transl. Sci..

[13] Pham E.C., Le Thi T.V., Le T.C., Nguyen T.M., Nguyen N.T. (2024). Dietary supplementation use for outpatient treatment in children: A cross-sectional study. Clin. Nutr. ESPEN.

[14] Lehrer B.J., Mutamba G., Thure K.A., Evans C.D., Hersh A.L., Banerjee R., Katz S.E. (2024). Optimal Pediatric Outpatient Antibiotic Prescribing. JAMA Netw. Open.

[15] Alkhaldi S.M., Yaseen N.A., Bataineh E.A., Al-Rawashdeh B., Albadaineh M.A., Mubarak S.M., Jaras R.E., Taha H.A. (2021). Patterns of antibiotic prescribing and appropriateness for respiratory tract infections in a teaching hospital in Jordan. Int. J. Clin. Pract..

[16] Mahmood R.K., Gillani S.W., Saeed M.W., Hafeez M.U., Gulam S.M. (2020). Systematic Review: Study of the Prescribing Pattern of Antibiotics in Outpatients and Emergency Departments in the Gulf Region. Front. Pharmacol..

[17] Alzahrani K.O., Alshahrani S.M., Alajel S.M. (2023). Evaluating the effectiveness of the Ministry of Health restriction policy on seasonal antibiotic consumption trends in Saudi Arabia, 2016–2020. Front. Pharmacol..

[18] STATA (2017). Stata Statistical Software: Release 16.

[19] Brown C.L., Montez K., Amati J.B., Simeonsson K., Townsend J.D., Orr C.J., Palakshappa D. (2021). Impact of COVID-19 on Pediatric Primary Care Visits at Four Academic Institutions in the Carolinas. Int. J. Environ. Res. Public Health.

[20] Casey S.D., Huang J., Parry D.D., Lieu T.A., Reed M.E. (2024). Health Care Utilization with Telemedicine and In-Person Visits in Pediatric Primary Care. JAMA Health Forum.

[21] Bazemore A., Petterson S., Peterson L.E., Phillips R.L. (2015). More Comprehensive Care Among Family Physicians is Associated with Lower Costs and Fewer Hospitalizations. Ann. Fam. Med..

[22] Balaguer Martínez J.V., del Castillo Aguas G., Gallego Iborra A. (2018). Antibiotics prescription and complementary tests based on frequency of use and loyalty in Primary Care. An. Pediatría.

[23] Ergül A.B., Gökçek İ., Çelik T., Torun Y.A. (2018). Assessment of inappropriate antibiotic use in pediatric patients: Point-prevalence study. Turk Pediatri Ars..

[24] Yehualaw A., Taferre C., Bantie A.T., Demsie D.G. (2021). Appropriateness and Pattern of Antibiotic Prescription in Pediatric Patients at Adigart General Hospital, Tigray, Ethiopia. BioMed Res. Int..

[25] Yan J., Hawes L., Turner L., Mazza D., Pearce C., Buttery J. (2018). Antimicrobial prescribing for children in primary care. J. Paediatr. Child Health.

[26] Ralston S.L., Lieberthal A.S., Meissner H.C., Alverson B.K., Baley J.E., Gadomski A.M., Johnson D.W., Light M.J., Maraqa N.F., Mendonca E.A. (2014). Clinical Practice Guideline: The Diagnosis, Management, and Prevention of Bronchiolitis. Pediatrics.

[27] Kimberlin D.W., Banerjee R., Barnett E.D., Lynfield R., Sawyer M.H., Committee on Infectious Diseases AAoP (2024). Red Book: 2024–2027 Report of the Committee on Infectious Diseases.

[28] de Bie S., Kaguelidou F., Verhamme K.M.C., De Ridder M., Picelli G., Straus S.M.J.M., Giaquinto C., Stricker B.H., Bielicki J., Sharland M. (2016). Using Prescription Patterns in Primary Care to Derive New Quality Indicators for Childhood Community Antibiotic Prescribing. Pediatr. Infect. Dis. J..

[29] El-Dahiyat F., Salah D., Alomari M., Elrefae A., Jairoun A.A. (2022). Antibiotic Prescribing Patterns for Outpatient Pediatrics at a Private Hospital in Abu Dhabi: A Clinical Audit Study. Antibiotics.

[30] Fleming-Dutra K.E., Hersh A.L., Shapiro D.J., Bartoces M., Enns E.A., File T.M., Finkelstein J.A., Gerber J.S., Hyun D.Y., Linder J.A. (2016). Prevalence of Inappropriate Antibiotic Prescriptions Among US Ambulatory Care Visits, 2010–2011. JAMA.

[31] Hersh A.L., Shapiro D.J., Pavia A.T., Shah S.S. (2011). Antibiotic prescribing in ambulatory pediatrics in the United States. Pediatrics.

[32] Sanchez G.V., Roberts R.M., Albert A.P., Johnson D.D., Hicks L.A. (2014). Effects of knowledge, attitudes, and practices of primary care providers on antibiotic selection, United States. Emerg. Infect. Dis..

[33] CDC (2022). Outpatient Antibiotic Prescribing in the United States: Centers for Disease Control and Prevention. https://www.cdc.gov/antibiotic-use/hcp/data-research/antibiotic-prescribing.html.

[34] Cabral C., Horwood J., Symonds J., Ingram J., Lucas P.J., Redmond N.M., Kai J., Hay A.D., Barnes R.K. (2019). Understanding the influence of parent-clinician communication on antibiotic prescribing for children with respiratory tract infections in primary care: A qualitative observational study using a conversation analysis approach. BMC Fam. Pract..

[35] Biezen R., Grando D., Mazza D., Brijnath B. (2019). Dissonant views—GPs’ and parents’ perspectives on antibiotic prescribing for young children with respiratory tract infections. BMC Fam. Pract..

[36] MoH (2024). Immunisation (Vaccines) Riyadh: Ministry of Health. https://www.moh.gov.sa/en/HealthAwareness/EducationalContent/vaccination/Pages/003.aspx.

[37] WHO (2016). WHO Guideline: Use of Multiple Micronutrient Powders for Point-Of-Use Fortification of Foods Consumed by Infants and Young Children Aged 6–23 Months and Children Aged 2–12 Years: World Health Organisation. https://www.who.int/publications/i/item/9789241549943.

[38] Alexander K.E., Brijnath B., Biezen R., Hampton K., Mazza D. (2017). Preventive healthcare for young children: A systematic review of interventions in primary care. Prev. Med..

[39] PHA (2022). Antimicrobial Resistance (AMR) Action Plan Kingdom of Saudi Arabia 2022–2025.

